# Computational Laser Spectroscopy in a Biological Tissue

**DOI:** 10.1155/2010/253763

**Published:** 2010-04-07

**Authors:** M. Gantri, H. Trabelsi, E. Sediki, R. Ben Salah

**Affiliations:** ^1^Unité de Rayonnement Thermique, Département de Physique, Faculté des Sciences de Tunis, 2092 EL Manar I, Tunisia; ^2^Unité de Biophysique, Faculté de Médecine de Sousse, Avenue Mohamed Karoui, 4002 Sousse, Tunisia

## Abstract

We present a numerical spectroscopic study of visible and infrared laser radiation in a biological tissue. We derive a solution of a general two-dimensional time dependent radiative transfer equation in a tissue-like medium. The used model is suitable for many situations especially when the external source is time-dependent or continuous. We use a control volume-discrete ordinate method associated with an implicit three-level second-order time differencing scheme. We consider a very thin rectangular biological-tissue-like medium submitted to a visible or a near infrared light sources. The RTE is solved for a set of different wavelength source. All sources are assumed to be monochromatic and collimated. The energetic fluence rate is computed at a set of detector points on the boundaries. According to the source type, we investigate either the steady-state or transient response of the medium. The used model is validated in the case of a heterogeneous tissue-like medium using referencing experimental results from the literature. Also, the developed model is used to study changes on transmitted light in a rat-liver tissue-like medium. Optical properties depend on the source wavelength and they are taken from the literature. In particular, light-transmission in the medium is studied for continuous wave and for short pulse.

## 1. Introduction

Diffuse light imaging and spectroscopy aims to investigate tissue physiology in subsurface. There is a spectral window existing within tissues in the near-infrared. The absorption of hemoglobin and water is small in the near-infrared, but elastic scattering from organelles and other microscopic interfaces is large. In tissue optics, any measuring is devoted for either spectroscopy or imaging. Spectroscopy is useful for measurement of time-dependent variations in the absorption and scattering of large tissue volumes. Many spectroscopic methods involving laser interactions in scattering media were employed to study different types of biological tissue [1–3]. Photon propagation in a biological tissue is affected by absorption and scattering. A computational light propagation model which describes the interaction of photons with scattering and absorbing media is essential in biomedical optics. It is useful for setting optical tissue properties and for the development of optical imaging algorithms [4–8]. 

The purpose of this paper is to produce a computational Laser spectroscopic study in a biological tissue. We aim to examine light-transmission through a biological tissue for different wavelength sources. Our concern is with modeling light propagation using the radiative transfer theory. We derive a model that unifies the basic applications of diffuse optical tomography which differ essentially by the time behavior of the external source. In particular, we will work with two choices for the external source, continuous wave and short-pulse. The simplest and easiest source to use is the continuous wave (CW). In this case, the source amplitude is constant, and the transmitted amplitude is measured as a function of source-detector separation or wavelength. The second type is a short-time-pulse. In this scheme, a short pulse is launched into the medium, and the temporal point spread function of the transmitted pulse is measured. First, we must say a few words about another way to model propagation of visible and near-infrared light in biological media which is the diffusion equation [9–11]. It is a limit behavior of the photon transport equation in a very high scattering media. However, low scattering and highly absorbing regions could not be accurately modeled by diffusion equation. Also, a forward model has to account for a hyperbolic propagation of the collimated laser pulse at the finite velocity of light in the medium, and diffusion approximations family fails to describe accurately this phenomenon [12]. For a general biological tissue, it would be more convenient to use a forward model based on radiative transfer equation (RTE). So, providing solutions to the RTE is a crucial research task especially in this domain. Concerning this problem, many works have used the RTE model with a variety of numerical techniques [13–17]. Most of these works are devoted to the numerical treatment of situations where a unique choice for the external source is considered. When the source is continuous the time-independent RTE is used. The supply of short-pulsed external source requires the use of a time-dependent RTE model. In all cases there, are many difficulties in implementing these models. To overcome some of these difficulties, many techniques of RTE-solution have been used either in time-independent or in time-dependent cases. Klose and Hielsher [18] have developed an upwind-difference discrete ordinate formulation of time-independent RTE. The authors tested their model with experimental data obtained from homogeneous tissue-phantoms and from phantoms that contain void-like regions. In another paper, Klose and Larsen [19] have used a simplified spherical harmonics equation to approximate the equation of radiative transfer for modeling light propagation in biological tissues. In that work, authors tried to demonstrate that the *S* *P*_*N*_ equation provides accurate solutions for a time-independent RTE. Boulanger and Charette [20] have used an upwind advection scheme to treat a propagation wave in homogenous and heterogeneous media submitted to a short-pulsed laser source. They proved that this technique can overcome a difficulty encountered in optical spectroscopy and due to high scattering regions especially in biological tissues. The most complete account of this problem is found in the works of Alfano group which has produced many papers in this field. Among these, we can cite papers [21, 22] where Cai et al. developed an analytical cumulant solution of the time-dependent radiative transfer equation in an infinite uniform medium with an arbitrary phase function. The same group of authors has made improvements in this approach especially by separating the ballistic component from the scattered component of light in [23, 24]. Such improvements allowed the authors to consider complicated situations like a polarized short pulse of light incident on a scattering medium. They even investigated backscattering of circularly polarized light from turbid media. Their theoretical results were validated by experimental data. Recently Pu et al. [25] investigated time-resolved and spectral measurements of the SW emission from cancerous and normal prostate tissues. This contributes to a more comprehension of Laser-tissues interactions and offers new fields to the radiative transfer application. 

In this work, we derive a control volume discrete-ordinate method associated with an implicit three-level second-order time differencing scheme to solve a two-dimensional time-dependent radiative transfer equation in a general biological tissue medium. We investigate many cases concerning the properties of the medium and the characteristics of the source. We will prove that the used model suits for all these applications. First, the proposed model is tested in the case of a heterogeneous tissue-like phantom illuminated by a continuous wave source. Experimental results are taken from [18]. Numerical results concerning the steady-state transmitted light on the boundary are compared with measurements. Next, the proposed model is used to establish a spectroscopic study with a continuous wave source. Results concerning transmitted light through a rat-liver tissue-like medium are presented for different wavelengths. Also, we present time-dependent spectroscopy for the same medium submitted to a one short-pulse. Results concerning transmitted light and internal light distribution are presented for some source wavelengths. Before all, theoretical and numerical background of our simulations is presented below.

## 2. General Time-Dependent RTE

### 2.1. Physical Model

Light propagation in a human biological tissue is modeled by using a general time-dependent RTE [26]:

(1) $$\begin{array}{cc} & \frac{n}{c}\frac{\partial I_{\lambda }\left(r,\Omega ,t\right)}{\partial t}+\Omega \cdot \nabla I_{\lambda }\left(r,\Omega ,t\right)+\left(\mu_{a}\left(r\right)+\mu_{s}\left(r\right)\right)I_{\lambda }\left(r,\Omega ,t\right)\\ & \;=\mu_{s}\int_{0}^{2\pi }p\left(\Omega ,\Omega^{\prime }\right)I_{\lambda }\left(r,\Omega^{\prime },t\right)d\Omega^{\prime }+S_{\lambda }\left(r,\Omega ,t\right). \end{array}$$

In this equation,

- *I*_*λ*_(**r**, **Ω**, *t*)  is the monochromatic directional energetic radiance at the spatial position-vector **r** = (*x*, *y*, *z*). When used in a dimensional form, the radiance *I*_*λ*_(**r**, **Ω**, *t*)  is often expressed in units of W·cm^−2^sr^−1^. The photon direction is described using a unitary direction vector  **Ω**;
- *μ*_a_(**r**)  and  *μ*_s_(**r**) are the absorption and the scattering coefficients, respectively. They are both given in units of cm^−1^. *c* is the speed of light in vacuum. *n* is the medium's refractive index. Also we make use of reduced scattering coefficient:
  (2) $$\mu_{\text{s}}^{\prime }=\left(1-\text{g}\right)\mu_{\text{s}};$$
- The source term *S*_*λ*_(**r**, **Ω**, *t*)  is an injected monochromatic radiance at the medium boundary;
- The phase function *p*(Ω, Ω′)  describes the probability that during a scattering event a photon with direction  **Ω**′ is scattered into the direction  **Ω**. In this paper, we use a commonly-applied phase function in tissue optics. It is the Henyey-Greenstein phase function [27, 28]:

(3) $$p\left(\cos\;\;\left(\theta \right)\right)=\frac{1-g^{2}}{4\pi {\left(1+g^{2}-2g\cos\;\left(\theta \right)\right)}^{2/3}},$$

where *g* is the anisotropy factor and *θ* is the angle between **Ω** and  **Ω**′. The integral of radiance over all angles **Ω** at one point **r** yields the fluence rate:

(4) $$\Phi_{\lambda }\left(r,t\right)=\int I_{\lambda }\left(r,\Omega ,t\right)d\Omega .$$

In order to lighten the equations, the subscript *λ* will be omitted in all the rest of this paper.

### 2.2. Boundary Condition

The radiance in the boundary is the sum of the external source contribution and the partly reflected radiance due to the refractive index mismatch. This gives

(5) $$I\left(r_{b},\Omega ,t\right)=S\left(r_{b},\Omega ,t\right)+R\cdot I\left(r_{b},\Omega_{\text{ref}},t\right),n\cdot \Omega <0\;,\Omega_{\text{ref}}\cdot n=-\Omega \cdot n,$$

where **r**_**b**_ is a vector position on the boundary and *n* is the unit outer normal vector. The reflectivity *R* is given for each direction using a Fresnel's relationship:

(6) $$\begin{array}{cc} R & =\frac{1}{2}{\left(\frac{n_{m\;}\cos\;\;\varphi_{\text{tra}}-n_{0}\cos\;\;\varphi_{\text{ref}}}{n_{m}\cos\;\;\varphi_{\text{tra}}+n_{0}\cos\;\;\varphi_{\text{ref}}}\right)}^{2}\\ & \;\;+\frac{1}{2}{\left(\frac{n_{0}\cos\;\;\varphi_{\text{ref}}\;-n_{m}\cos\;\;\varphi_{\text{tra}}}{n_{0}\cos\;\;\varphi_{\text{ref}}\;+n_{m}\cos\;\;\varphi_{\text{tra}}}\right)}^{2}\\ & \;\;\text{if}\;\varphi_{\text{ref}}<\varphi_{\text{cr}}\;\text{and}\;\text{else},\;R=1, \end{array}\;$$

where *φ*_ref_  and *φ*_tra_ are the reflection and the transmission angle respectively. In the air, the transmission angles, is calculated through the following Snell's law:

(7) $$n_{m}{\sin\varphi }_{\text{ref}}=n_{0}{\sin\varphi }_{\text{tra}}.$$

The medium refractive index *n*_*m*_ depends on the tissue and *n*_0_ is taken as unity. The critical angle is determined by the following equation:

(8) $${\sin\varphi }_{\text{cr}}=\frac{n_{0}}{n_{m}}.$$

Thus, the transmitted fluence rate at the boundary can be computed in each detector point using the following integral formula:

(9) $$\Phi_{\text{tra}}\left(r_{b},t\right)=\int_{\Omega \cdot n>0}^{}\left(1-R\right)I\left(r_{b},\Omega ,t\right)d\Omega .$$

The fluence rate on the boundary which enters the detector for a given aperture AP is computed as.

(10) $$\Phi_{d}\left(r_{b},t\right)=\int_{\text{Ap}}^{}\left(1-R\right)I\left(r_{b},\Omega ,t\right)d\Omega .$$

In this work, Φ_*d*_ will be called detected fluence rate and we will take AP = 45° in all our investigations.

## 3. Solution Method and Numerical Implementation

### 3.1. Control Volume Discrete Ordinates Method: CVDOM

In this paper, we use a control volume discrete-ordinate method [29, 30] associated with an implicit three-level second-order time differencing scheme to solve (1) in a general biological tissue medium. At first, this requires the integration of (1) on an elementary control volume noted ∆*V*. The spatial calculus domain is divided into a set of *I* × *J* elementary uniform volumes ∆*V* with unitary depth. This yields a calculus domain subdivided in *I* ∗ *J* rectangular cells. Each (*i*, *j*)-cell is Δ*x*∆*y*-sized. So, we can write.

(11) $$\begin{array}{cc} \frac{n}{c}\frac{\partial I_{P,m}}{\partial t}+{\Delta y\cdot \xi }_{m}\left(I_{E,m}-I_{W,m}\right)\\ & \;+{\Delta x\cdot \eta }_{m}\left(I_{N,m}-I_{S,m}\right)+\Delta x\cdot \Delta y\left(\mu_{a}+\mu_{s}\right)I_{P,m}\\ & =\Delta x\cdot \Delta y\left(S_{P,m}+\mu_{s}\overset{M}{\underset{m^{\prime }=1}{\sum }}w_{m^{\prime }}{p_{m,m^{\prime }}I}_{P,m^{\prime }}\right), \end{array}$$

where *ξ*_*m*_ = cos(****Ω****_*m*_ · *n^x^*) and *η*_*m*_ = cos(****Ω****_*m*_ · *n^y^*) with *n*_*x*_  and  *n*_*y*_ being two normal vectors to the (*x*, *z*)-plane and the (*y*, *z*)-plane, respectively. *w*_*m*_ is a weighting factor depending on the chosen quadrature formula. In this work, we use a constant weight quadrature. After angular discretisation, the phase function term becomes

(12) $$p_{mm^{\prime }}=\frac{1-g²}{4\pi {\left(1+g²-2g\left(\xi_{m}\xi_{m^{\prime }}+\eta_{m}\eta_{m^{\prime }}\right)\right)}^{3/2}}.$$

If the direction cosines are positive, the directional energetic radiances are known on the faces *W* and *S* and they are unknown on the faces *E* and *N* of the (*i*, *j*)-cell and also in the centre *P*. Therefore, we need two complementary relations to eliminate *I*_*E*,*m*_ and *I*_*N*,*m*_.These relations can be obtained by using interpolation formula:

(13) IP,m=α  IE,m+(1−α)  IW,m,IP,m=α  IN,m+(1−α)  IS,m,

where *α* is an interpolation parameter. Using the above formulae, (11) can be written as

(14) $$\begin{array}{cc} & \frac{n}{c}\frac{\partial I_{P,m}}{\partial t}+\frac{{\Delta y\cdot \xi }_{m}}{\alpha }\left(I_{P,m}-I_{W,m}\right)+\frac{{\Delta x\cdot \eta }_{m}}{\alpha }\left(I_{P,m}-I_{S,m}\right)\\ & \;+\Delta x\cdot \Delta y\left(\mu_{a}+\mu_{s}\right)I_{P,m}\\ & =\Delta x\cdot \Delta y\left(S_{P,m}+\mu_{s}\overset{M}{\underset{m^{\prime }=1}{\sum }}w_{m^{\prime }}{p_{m,m^{\prime }}I}_{P,m^{\prime }}\right). \end{array}$$

For time discretization, an implicit three-level second-order time differencing scheme [31] is used:

(15) $$\frac{\partial I_{P,m}}{\partial t}=\frac{3I_{P,m}^{n+1}-4I_{P,m}^{n}+I_{P,m}^{n-1}}{2∆t},\;n=1,\ldots n_{\max\;},$$

whith Δ*t* being the discrete time step. So we can rewrite (14) as follows:

(16) $$\begin{array}{cc} & I_{P,m}^{n+1}\left(\frac{3n}{2c∆t}+\frac{{\Delta y\cdot \xi }_{m}}{\alpha }+\frac{{\Delta x\cdot \eta }_{m}}{\alpha }+\Delta x\cdot \Delta y\left(\mu_{a}+\mu_{s}\right)\right)\\ & \;=\frac{4n}{2c∆t}I_{P,m}^{n}-\frac{n}{2c∆t}I_{P,m}^{n-1}+\frac{{\Delta y\cdot \xi }_{m}}{\alpha }I_{W,m}^{n+1}+\frac{{\Delta x\cdot \eta }_{m}}{\alpha }I_{S,m}^{n+1}\\ & \;\;+\Delta x\cdot \Delta y\left(S_{P,m}+\mu_{s}\overset{M}{\underset{m^{\prime }=1}{\sum }}w_{m\prime }p_{m,m\prime }I_{P,m\prime }^{n+1}\right).\; \end{array}$$

Theoretically, if we know *I*_*P*,*m*_ on the (*i*, *j*)-cell, we can obtain results over the cells (*i* + 1, *j*) and (*i*, *j* + 1) by using the boundary conditions and the following relations:

(17) IW,m(i+1,j)=IE,m(i,j), i=1,…,I−1,IS,m(i,j+1)=IN,m(i,j), j=1,…,J−1.

If (*ξ*_*m*_ > 0  and  *η*_*m*_ > 0), then we get the following equation:

(18) $$\begin{array}{cc} I_{i,j,m}^{n+1} & =\;{\left[\frac{3n}{2c∆t}+\frac{{\Delta y\cdot \xi }_{m}}{\alpha }+\frac{{\Delta x\cdot \eta }_{m}}{\alpha }+\Delta x\cdot \Delta y\left(\mu_{a}+\mu_{s}\right)\right]}^{-1}\\ & \;\times [\frac{4n}{2c∆t}I_{i,j,m}^{n}-\frac{n}{2c∆t}I_{i,j,m}^{n-1}+\frac{{\Delta y\cdot \xi }_{m}}{\alpha }I_{i-1,j,m}^{n+1}\\ & \;\;\;+\frac{{\Delta x\cdot \eta }_{m}}{\alpha }I_{i,j-1,m}^{n+1}+\Delta x\\ & \;\;\;\cdot \Delta y\left(S_{i,j,m}+\mu_{s}\overset{M}{\underset{m^{\prime }=1}{\sum }}w_{m^{\prime }}p_{m,m^{\prime }}I_{i,j,m^{\prime }}^{n+1}\right)]. \end{array}$$

In (18), we have used these notations:

- *I*_*i*,*j*,*m*_^*n*^ = *I*(*i*Δ*x*, *j*Δ*y*, *ξ*_*m*_, *η*_*m*_, *n*Δ*t*)
- *S*_*i*,*j*,*m*_^*n*^ = *S*(*i*Δ*x*, *j*Δ*y*, *ξ*_*m*_, *η*_*m*_, *n*Δ*t*).

To solve (18), we use successive iterations to actualise the implicit internal source term in the right member. So, we obtain for  *n* ≥ 1,

(19) $$\begin{array}{cc} & {\left(I_{i,j,m}^{n+1}\right)}^{k+1}\\ & \;={\left[\frac{3n}{2c∆t}+\frac{{\Delta y\cdot \xi }_{m}}{\alpha }+\frac{{\Delta x\cdot \eta }_{m}}{\alpha }+\Delta x\cdot \Delta y\left(\mu_{a}+\mu_{s}\right)\right]}^{-1}\\ & \;\;\;\times [\frac{4n}{2c∆t}\overset{®}{I_{i,j,m}^{n}}-\frac{n}{2c∆t}\overset{®}{I_{i,j,m}^{n-1}}+\frac{{\Delta y\cdot \xi }_{m}}{\alpha }{\left(I_{i-1,j,m}^{n+1}\right)}^{k+1}\\ & \;\;\;\;+\frac{{\Delta x\cdot \eta }_{m}}{\alpha }{\left(I_{i,j-1,m}^{n+1}\right)}^{k+1}\\ & \;\;\;\;+\Delta x\cdot \Delta y\left(S_{i,j,m}^{n+1}+\mu_{s}\overset{M}{\underset{m^{\prime }=1}{\sum }}w_{m^{\prime }}p_{m,m^{\prime }}{\left(I_{i,j,m^{\prime }}^{n+1}\right)}^{k}\right)]. \end{array}$$

The iteration process is repeated until a convergence criterion is attempted and to improve convergence speed, we use a successive over relaxation technique [32]. So the updated value (*I*_*i*,*j*,*m*_^*n*^)_updated_^*k*+1^ becomes a linear combination of the iterated value (*I*_*i*,*j*,*m*_^*n*^)^*k*+1^ and the previously computed value (*I*_*i*,*j*,*m*_^*n*^)^*k*^ as

(20) $${\left(I_{i,j,m}^{n}\right)}_{\text{updated}}^{k+1}=\left(1-\rho \right){\left(I_{i,j,m}^{n}\right)}^{k}+\rho \;{\left(I_{i,j,m}^{n}\right)}^{k+1},$$

where *ρ* being a relaxation parameter whose value is usually between 1 and 2. In most of our calculus, we have used: *ρ* = 1.1. The solution is obtained when the relative discrepancy value, *ϵ* = |(*I*_*i*,*j*,*m*_^*n*^)^*k*+1^ − (*I*_*i*,*j*,*m*_^*n*^)^*k*^/(*I*_*i*,*j*,*m*_^*n*^)^*k*^|, is smaller than a tolerance value tol. In that case, we take (*I*_*i*,*j*,*m*_^*n*^)^*k*^ as a solution. It will be noted ${\overset{®}{I}}_{i,j,m}^{n}$. 

It is worth noting that, in all our simulations, we take a tolerance value: tol = 10^−8^ and for initial condition, we take ${\overset{®}{I}}_{i,j,m}^{0}=0.$ We use Δ*x* = Δ*y* and a uniform time step Δ*t* = 10^−13^s. Also it is important to mention that when a continuous source is injected, another iterating process is implemented to attempt the steady-state solution. This is obtained by iterating the solution ${\overset{®}{I}}_{i,j,m}^{n}$ until$\left|{\overset{®}{I}}_{i,j,m}^{n}-{\overset{®}{I}}_{i,j,m}^{n-1}/{\overset{®}{I}}_{i,j,m}^{n-1}\right|\leq \text{tol}.$ At the equilibrium, all time derivatives will be vanished and we will get a solution of the time-independent radiative transfer equation. This procedure could be a validation criterion for the implemented algorithm convergence. Now, if the direction cosines are not positive, the precedent equations are valid provided that the orientation WESN of cells is done according to the direction of propagation. To sweep the calculus domain, we use negative increments. So, the set of all angular directions (**Ω**_*m*_)  is divided into four subsets depending on the sign of *ξ*_*m*_ and *η*_*m*_. By the use of direction cosine sign, all cases can be given through the following general equation:

(21) $$\begin{array}{cc} & {\left(I_{i,j,m}^{n+1}\right)}^{k+1}\\ & \;=\;[\frac{3n}{2c∆t}+\frac{\Delta y\cdot \mathrm{sign}\;\left(\xi_{m}\right)\xi_{m}}{\alpha }\\ & \;\;\;{+\frac{\Delta x\cdot \mathrm{sign}\;\left(\eta_{m}\right)\eta_{m}}{\alpha }+\Delta x\cdot \Delta y\left(\mu_{a}+\mu_{s}\right)]}^{-1}\\ & \;\;\times [\frac{4n}{2c∆t}\overset{®}{I_{i,j,m}^{n}}-\frac{n}{2c∆t}\overset{®}{I_{i,j,m}^{n-1}}\\ & \;\;\;\;+\frac{\Delta y\cdot \mathrm{sign}\;\left(\xi_{m}\right)\xi_{m}}{\alpha }\cdot {\left(I_{i-1\times \mathrm{sign}\;\left(\xi_{m}\right),j,m}^{n+1}\right)}^{k+1}\\ & \;\;\;\;+\frac{\Delta x\cdot \mathrm{sign}\;\left(\eta_{m}\right)\eta_{m}}{\alpha }{\left(I_{i,j-1\times \mathrm{sign}\;\left(\eta_{m}\right),m}^{n+1}\right)}^{k+1}\\ & \;\;\;\;+\Delta x\cdot \Delta y\left(S_{i,j,m}^{n+1}+\mu_{s}\overset{M}{\underset{m^{\prime }=1}{\sum }}w_{m^{\prime }}p_{m,m^{\prime }}{\left(I_{i,j,m^{\prime }}^{n+1}\right)}^{k}\right)]. \end{array}$$

### 3.2. External Source and Computed Quantities

In all our investigations, the power laser-source is assumed to be equivalent to a forward collimated radiance injected at a pinpoint on the bottom side of the boundary. We use two types of source:

- a continuous wave source with a uniform equivalent intensity value of 20 mWcm^−2^sr^−1^.
- a short-Laser-pulse source, applied only at the first instant of calculus. It is a 100fs-pulse having an equivalent radiance value of 200 mWcm^−2^sr^−1^,

In our presented results on the boundary, we use either calculated detected fluence rate or normalized detected fluence rate:

(22) $$\Phi_{d}^{N}=\frac{\phi_{d}}{1/D\sum_{d}^{D}{\;w_{d}\phi }_{d}},$$

where  *ϕ*_*d*_is the computed detected fluence rate at a detector point *d*, *D* is the number of the detector points on the concerned side of the boundary, and *w*_*d*_ is a weighting factor from the generalized trapezoidal integration rule [34]. Also, distribution of light in the medium is presented through the calculated fluence rate matrix which is given at the instant, *n*Δ*t*, as:

(23) $$\phi_{ij}^{n}=\overset{M}{\underset{m=1}{\sum }}w_{m}{\overset{®}{I}}_{i,j,m}^{n}.$$

****

## 4. Results and Discussion

### 4.1. Model Validation

**Figure 1:**
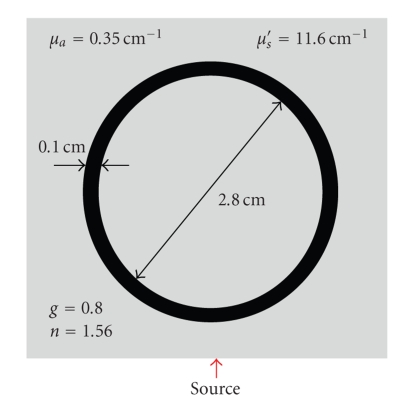
Geometry and optical properties of the referencing medium [18].

To test our model, we have considered a case of 678 nm-light punctual continuous wave source. Our Model is tested by comparing obtained results with phantom experimental data reported in [18]. Figure 1 shows the phantom, it is assumed to be 4 cm × 4 cm sized heterogeneous medium which contains a water-filled, void-like ring with an inner diameter of 2.8 cm and an outer diameter of 3.0 cm. A continuous source is placed on the middle of the bottom side of the boundary. The numerically predicted results are compared with corresponding experimentally measured data extracted from in [18, Figure  7]. The convergence of our algorithm is conditioned by some considerations in grid-mesh characteristics. In particular, the choice of the cell dimension should be taken too small. More precisely, to move from a cell to another neighboring cell, we should travel less than the total mean free path of photons. So, it should be more convenient to do calculations under the condition: Δ*x* < 1/(*μ*_*a*_ + *μ*_*s*_′). In these calculations, we have used Δ*x* = Δ*y* = 0.05 cm.

**Figure 2:**
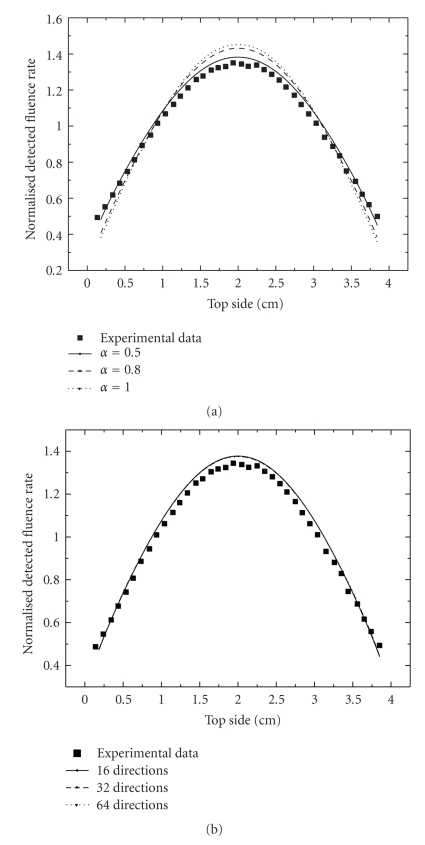
Comparison of calculated normalized steady state transmitted fluence rate and experimental data of a heterogeneous tissue-like medium. Calculus is done in the case of a continuous wave source for different values of the interpolation parameter (a) and for different angular grids (b).

Figure 2(a) represents the calculated steady state normalized transmitted fluence rate on the opposite side to the source for different values of the interpolation parameter. The case (*α* = 0.5) shows the closest results to the experimental measurements. In the rest of investigations, the interpolation parameter value will be putted 0.5. Also Figure 2(b) shows an almost angular grid-independency of our numerical results. We will take a set of 16 uniformly distributed discrete angles which is sufficient to do the rest of our calculus. These results and other results shown in [14] constitute both numerical tests and experimental validations of our model. 

### 4.2. Computational Spectroscopy with a Continuous Wave Source

**Figure 3:**
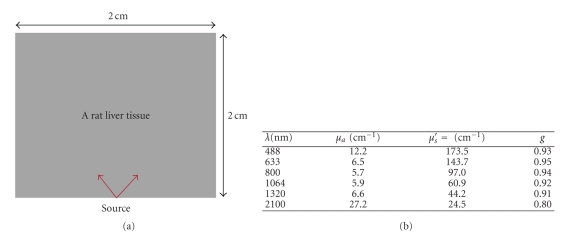
Geometry and optical properties of the rat-liver-tissue at different wavelengths [33].

**Figure 4:**
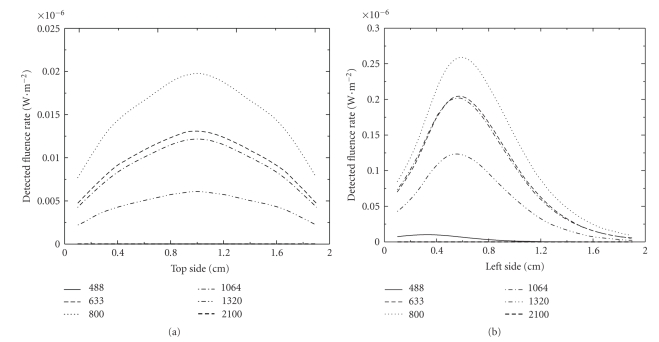
Detected fluence rate for different source-wavelengths. Calculus is done in the case of a continuous wave source. Results are shown for top side (a) and left side (b).

**Figure 5:**
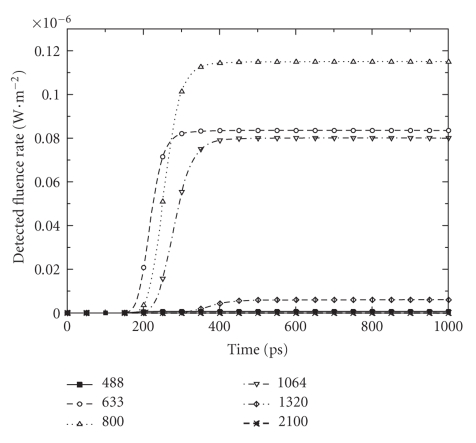
Time-dependent detected fluence rate at a detector point on top side of the rat-liver tissue-like medium. Calculus is done in the case of a continuous wave source.

In this investigation, we consider a rat liver tissue-like medium as it is shown in Figure 3. Optical properties of the tissue are taken from [33] from a set of several wavelengths. Figures 4(a) and 4(b) illustrate transmission through the considered medium on the top side and on the left side for different laser-source wavelengths. Calculus is carried out as in the previous paragraph for a continuous wave source. Transmitted directional radiances are calculated in 18 detector points on the top side and on the left side of the boundary. Figures 4(a) and 4(b) show that transmission for 488 nm and 2100 nm is very weak. The maximal transmission from the tissue is observed in the red and the near infrared part of radiation spectrum. Also, Figure 5 shows the time behavior of the detected fluence rate in a point on the top side of the studied medium. During the transient period, Figure 4 shows no response of the medium for 488 nm and 2100 nm and a very weak transmission in 1320 nm and a relative larger global transmission in red and near infrared zones. 

### 4.3. Spectroscopy with a Short-Laser Pulse

**Figure 6:**
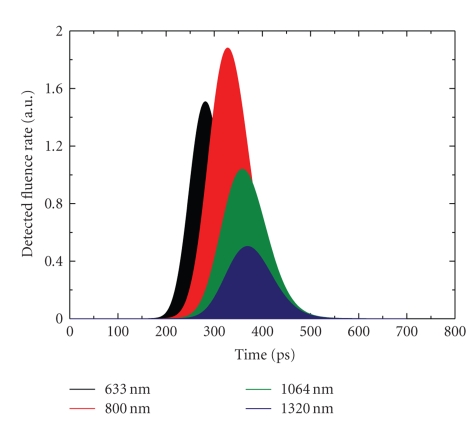
Time-dependent detected fluence rate on the top side of the rat-liver tissue-like medium. Calculus is done in the case of a 100fs-laser pulse injected in the bottom side with different wavelengths.

**Figure 7:**
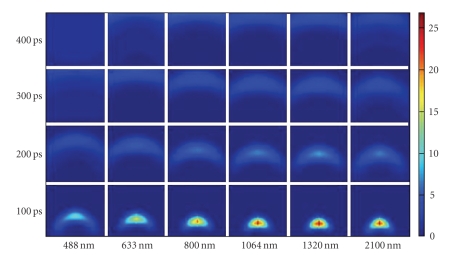
Internal light distribution in the rat-liver tissue-like medium for different instants after the pulse. Calculus is done in the case of a 100fs-laser pulse for different wavelengths.

 In this investigation, we study the same medium as in the just precedent paragraph but we use a short-pulsed source. In all cases, it is a 100fs-laser pulse injected in the medium through a point on the middle of the bottom side of the boundary. Figure 6 shows the transmitted light at a detector point on the top side of the boundary. Results are presented for a set of different wavelengths. Only near infrared light displays a relative appreciable transmission. A maximum of detected fluence rate is noted at 800 nm-light. A little delay of the maximum detection of radiation is observed when wavelength increases. Figure 7 shows fluence rate distribution into the medium at different moments after the pulse. In all moments, symmetric propagation of light is observed into the medium. Also, it can be observed that scattering prevails in 488 nm while more and more absorption is observed in 2100 nm. At 488 nm, the phenomenon of multiple scattering is dominant into the medium. The dispersion of the pulse is accentuated in all sides. Light disappears after 250ps. At 2100 nm, more light is absorbed into the medium so a fraction of energy persists into the medium for more time. 

**Figure 8:**
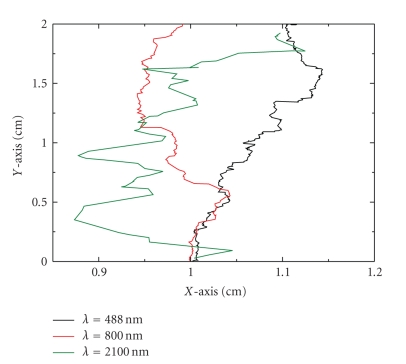
Typical trajectory of photons for different values of the wavelength source as obtained through Monte Carlo simulations.

Figure 8 shows typical movement of different photons in the medium at three different wavelengths. Calculations in our studied medium are carried out by using Monte Carlo simulations as it is described in [35]. It can be observed that a typical 800 nm-photon is almost snake. For 488 nm-photon, the mean scattering free path is very small. Multiple scattering is dominant. The typical trajectory in this case is not ballistic and the photons are almost diffusive. For 2100 nm, the mean scattering free path is longer than the 488 nm-photon but the absorption mean free path is narrower. A typical photon in this wavelength is almost absorbed into the medium. 

## 5. Conclusion

This paper investigated situations concerning biological tissue interaction with Laser radiation by using radiative transfer theory. More precisely, a computational radiative transfer model is used to establish a numerical Laser spectroscopic study in a rat-liver-tissue-like medium. The response of the medium to both time-dependent and time-independent sources is explored. Obtained Results are tested by using experimental measurements from the literature and by using Monte Carlo simulations. The maximal transmission from the tissue is observed in the red and the near infrared part of radiation spectrum. Also multiple scattering is prevailing in 488 nm while more and more absorption is observed in 2100 nm.

## Nomenclature

*c*: Speed of light, (in vacuum), cm/s
*g*: Anisotropy factor
*I*: Intensity, W · cm^−2^ *sr* ^−1^
*n*: Refractive index
*p*(Ω, Ω′): Phase function
**r**: Position vector, cm
*R*: Reflectivity coefficient
**r**_**b**_: Position vector in the boundary
*S*: Source term, W · cm^−3^ *sr* ^−1^
*w*_*d*_: Weighting factor from trapezoidal integration
*w*_*m*_: Weight associated with discrete direction
*x*, *y*: Cartesian coordinates, cm.

## Greek Symbols

*α*: Interpolation parameter
Φ: Fluence rate, W · cm^−2^
Φ_*d*_: Detected fluence rate, W · cm^−2^
*λ*: Wavelength, nm
Ω: Direction solid angle, *sr*
*μ*_*a*_: Absorption coefficient, cm^−1^
*μ*_*s*_: Scattering coefficient, cm^−1^
*μ*_*s*_′: Reduced scattering coefficient, cm^−1^
*ρ*: Relaxation parameter
*θ*: Scattering angle, rad
*ξ*, *η*: Direction cosines.
